# Fabrication and evaluation of the regenerative effect of a polycaprolactone/chitosan nanofibrous scaffold containing bentonite nanoparticles in a rat model of deep second-degree burn injury

**DOI:** 10.22038/IJBMS.2023.69930.15210

**Published:** 2024

**Authors:** Seyedeh-Sara Hashemi, Ali-Akbar Mohammadi, Mehdi Kian, Alireza Rafati, Mojtaba Ghaedi, Behzad Ghafari

**Affiliations:** 1 Burn and Wound Healing Research Center, Shiraz University of Medical Sciences, Shiraz, Iran; 2 Division of Plastic and Reconstructive Surgery, Department of Surgery, Shiraz University of Medical Sciences, Shiraz, Fars, Iran; 3 Department of Comparative Biomedical Sciences, School of Advanced Medical Sciences and Technologies, Shiraz University of Medical Sciences, Shiraz, Fars, Iran; 4 Student Research Committee, Shiraz University of Medical Sciences, Shiraz, Fars, Iran; 5 Division of Pharmacology and Pharmaceutical Chemistry, Sarvestan Branch, Islamic Azad University, Sarvestan, Fars, Iran; 6 Department of Surgery, School of Medicine, Jahrom University of Medical Sciences, Jahrom, Fars, Iran

**Keywords:** Bentonite-nanoparticles, Nanofibrous scaffold, Polycaprolactone, Rat, Wound healing

## Abstract

**Objective(s):**

In the present study, we evaluated the effect of a nanofibrous scaffold including polycaprolactone (PCL), chitosan (CHT), and bentonite nanoparticles (Ben-NPS) on wound healing in order to introduce a novel dressing for burn wounds.

**Materials and Methods:**

PCL, PCL/CHT, and PCL/CHT/Ben-NPS nanofibrous scaffolds were fabricated by the electrospinning technique. Their structural and physiochemical characteristics were investigated by Fourier-transform infrared spectroscopy (FTIR) analysis, scanning electron microscopy (SEM), tensile strength, water contact angle, as well as, swelling and degradation profiles test. The disc diffusion assay was carried out to investigate the antibacterial potential of the scaffolds. In addition, the cell viability and proliferation ability of human dermal fibroblasts (HDFs) on the scaffolds were assessed using MTT assay as well as SEM imaging. The wound-healing property of the nanofibrous scaffolds was evaluated by histopathological investigations during 3,7, and 14 days in a rat model of burn wounds.

**Results:**

SEM showed that all scaffolds had three-dimensional, beadles-integrated structures. Adding Ben-NPS into the PCL/CHT polymeric composite significantly enhanced the mechanical, swelling, and antibacterial properties. HDFs had the most cell viability and proliferation values on the PCL/CHT/Ben-NPS scaffold. Histopathological evaluation in the rat model revealed that dressing animal wounds with the PCL/CHT/Ben-NPS scaffold promotes wound healing.

**Conclusion:**

The PCL/CHT/Ben-NPS scaffold has promising regenerative properties for accelerating skin wound healing.

## Introduction

Skin is one of the highly susceptible organs in the body to various injuries, such as trauma, abrasion, surgery, diabetes, and particularly burn damage. After the skin damage, the wound healing process immediately initiates to recover the normal skin tissue’s structure and function (1, 2). However, this process can be restricted by the lack of adequate supplies of autologous skin grafts or donor sites which causes skin wounds to be at risk of some complications such as the formation of hypertrophic scar (excessive production of granulation tissue), keloid (overproduction of collagen), as well as wound dehiscence (physical rupture) and wound contractures (3, 4). Burn lesions ensue from exposure of the skin to heat, chemicals, electricity, or radiation. In deep or widespread burns, several life-threatening complications such as bacterial sepsis and hypovolemic shocks can consequently occur. The formation of scarring tissue is one of the most common consequences of burn injury. While there are several therapeutic approaches for healing burn wounds, they often have moderate effects. Therefore, more efficacious treatments to heal burn wounds are still needed (5). 

Wound dressing using biomaterial-based scaffolds is an emerging therapeutical strategy for enhancing tissue regeneration as well as the healing process in skin injuries such as burn wounds. The principal notion in the application of biomaterial-based scaffolds for wound dressing is to imitate the extracellular matrix (ECM) of the tissue (1, 6, 7). A good wound dressing scaffold should maintain moisture, absorb exudate, and inhibit bacterial activities in the wound sites. To achieve these goals, many polymeric scaffolds with natural or/and synthetic origins have been fabricated and experimentally examined (6). Polycaprolactone (PCL) and chitosan (CHT) are common polymeric biomaterials that are extensively used in wound dressing. 

PCL is a semi-crystalline synthetic aliphatic polyester that is commonly used as a wound dressing biomaterial. Although PCL possesses many favorable properties such as cost-effectiveness, availability, stability, biodegradability, and high mechanical resistance, also, it is a hydrophobic biomaterial without cell recognition sites, which leads to poor cell adhesion on the scaffold (8). Hence, such synthetic polymers usually blend with natural biomaterials. CHT is an aminated polysaccharide derived from chitin. CHT is a natural, non-toxic, biocompatible, and biodegradable, polymer that has antimicrobial activity (9, 10). Hence, it is commonly used in the composite biomaterials that are fabricated for tissue engineering (11). Bentonite (Ben) is an absorbent aluminum phyllosilicate clay that contains montmorillonites. Ben is shown to act efficiently in the healing of skin lesions and ulcers (10, 12, 13). It has been proved that Ben reinforced both PCL (14) and CHT (15, 16) scaffolds, but they were not used altogether.

Electrospinning is a suitable, simple, and affordable method that has the ability to provide nanoscale fibers with various physical, chemical, and biological attributes. The scaffolds produced with the electrospinning method can possess a well-conformable mesh structure with a desirable microenvironment for cellular infiltration and subsequent proliferation as well as tissue integration. Hitherto, many nanofibrous scaffolds composed of natural, synthetic, or semisynthetic biomaterials have been made and applied in skin tissue engineering which exhibits promising potential in the healing of wounds and prevention of scar formation (17-19). 

In the present study, an electrospun nanofibrous scaffold containing PCL, CHT, and Ben nanoparticles (-NPS) was developed in order to dress burn wounds. The morphology and biocompatibility of the PCL/CHT/Ben-NPS scaffold and the capacity of cell proliferation on it were assessed *in vitro*. Besides, the potential impact of the PCL/CHT/Ben-NPS scaffold on wound dressing was examined in a rat model of a deep partial-thickness burn.

## Materials and Methods

***Chemicals***

PCL, CHT (high molecular), Ben, Fetal Bovine Serum (FBS), Amphotericin B (~80%), Ethanol (≥99.8%), Dimethyl Sulfoxide (DMSO), Thiazolyl Blue Tetrazolium Bromide (98%), Phosphate buffer saline (PBS, Tablet), Dispase® I, Formaldehyde solution, Glutaraldehyde solution, Trypan Blue, Trypsin (lyophilized powder), and penicillin/streptomycin (100X, Tissue Culture Grade) were acquired from Sigma-Aldrich (St. Louis, MO, USA). Glacial acetic acid (≥99.5-100.5%) was obtained from Arman-Sina Chemical & Pharmaceutical Co. (Tehran, Iran). 

***Fabrication of fibrous scaffolds***

3D scaffolds were fabricated using the electrospinning technique. After optimization, 1.8 mg of PCL pellets were dissolved in 10 ml glacial acid acetic. Also, 0.04 g of CHT powder and 100 µg/ml of Ben-NPS solution were separately dissolved in 30% acetic acid. The solutions were all made at 50 °C for an hour while being magnetically stirred. For electrospinning, 18% PCL solution, as well as blend solutions of PCL/CHT at the ratio of 3:1 and PCL/CHT/Ben-NPS at the ratio of 3:1:1, were prepared separately. In order to improve homogeneity, the blend solutions were re-homogenized using a magnetic stirrer. After that, the PCL/CHT/Ben-NPS blend was loaded into a syringe pump and a nanofibrous scaffold was created by applying a voltage of 16 kV employing an electrospinning machine (Fanavaran Nano Meghyas Co. Ltd., Tehran, Iran). There was about a 14 cm distance between the collector plate and the nozzle (20, 21).

***Scanning electron microscopy (SEM) and imageJ characterization***

SEM was performed by a Vega3 Tescan microscope (Czech Republic) to examine the microstructure and the quality of the fabricated fibrous scaffolds. Afterward, SEM images of the scaffolds were analyzed using a plugin of ImageJ, DiameterJ, for parameters including fiber radius frequency, intersection density, number of pores, pore area, and porosity according to previous studies (22-25).

***FTIR spectroscopy analysis***

FTIR spectroscopy was conducted to characterize the chemical composition of the PCL, PCL/CHT, and PCL/CHT fibers using a spectrophotometer (RX1 Spectrum model, Perkin Elmer). For this purpose, first, KBr powder was used to grind and pelletize the scaffolds. The equipment was run at a resolution of 4 cm^-1^, and the IR spectra were then investigated between 400 and 4000 cm^-1^.

***Swelling behavior***

To investigate the swelling behavior of the fibrous scaffolds, the initial weight of three dry samples (1 cm×1 cm) from each scaffold was measured. Afterward, the samples were put into 15 ml tubes, immersed in 10 ml PBS, and placed in a 37 °C oven. At 1, 6, 12, and 24 hr, the samples were removed from the tubes and their weight was recorded following drying their surface water with a filter paper. Eventually, the samples were weighed, and Eq. 1 was used to calculate the swelling ratio. 

Swelling ratio=Final weight-Initial Weight Initial Weight×100

 (1)

***In vitro degradation assay***

Three dry samples (1 cm x 1 cm) from each fibrous scaffold were weighed, placed in 15 ml tubes with 10 ml PBS, and incubated at 37 °C for 1, 3, 5, 7, 14, and 21 days to determine how quickly the scaffolds degraded. Samples were taken out of PBS at each interval and allowed to air dry. The dried sample weight was then calculated. For each time point, distinct samples were utilized. The remaining weight ratio of each sample was calculated by Eq. 2.

$\mathrm{Remaining Weight}\left(\%\right)=(W_{i}-W_{r})/W_{i}\times 100$

 (2)

Where W_i_ and W_r_ are the initiating and remaining weight, respectively.

***Water contact angle evaluation***

The surface hydrophilicity of the PCL, PCL/CHT, and PCL/CHT/Ben-NPS fibrous scaffolds was assessed using the sessile drop method (26). Three drops of distilled water were placed on each mat before it was placed on a support holder. After placement, the images of the droplets were captured (SSC, DC318P color video camera, Japan), and the water contact angle was measured using ImageJ software (version 1.51w, NIH, USA) (20). 

***Mechanical characterization***

For evaluating the mechanical characteristics of PCL, PCL/CHT, and PCL/CHT/Ben-NPS fibrous scaffolds, samples were firstly cut into strips (50 × 10 mm) and pulled at the rate of 6 mm/min using a universal testing machine (STM-20 model, SANTAM, Iran). At least three samples were tested for each fibrous composite scaffold. The resulting stress-strain curves were used to calculate ultimate tensile strength (UTS) and elongation at break. 

***Isolation and culture of fibroblasts***

Human dermal fibroblasts (HDFs) were obtained from human foreskin samples according to previous studies (9). Briefly, PBS containing 1% penicillin/streptomycin was used to wash the samples, and hippodrome was detached using a sterilized surgical blade and then the epidermis layer was separated from the dermis. Cells from the dermis were then removed and cultivated in complete cell culture medium and kept at 37 °C and 5% CO_2_. Every three days, the cells were passaged.

***SEM for proliferation and cell adhesion***

The circular samples from the PCL, PCL/CHT, and PCL/CHT/Ben-NPS fibrous scaffolds were prepared to fit well in a 96-well plate. After the sterilization of both sides of the samples, they were placed into 96-well plates, and each well received 1×10^4^ HDFs before being incubated at 37 °C. The scaffolds were subsequently fixed with 2.5% glutaraldehyde for 4 hr at 4 °C, dehydrated progressively with ethanol concentrations of 25%, 50%, 70%, 95%, and 100% v/v, and examined by SEM to assess cell adhesion and proliferation. ImageJ watershed algorithm was used to count the number of cells on the electrospun scaffolds.

***MTT assay***

The MTT test was used to evaluate the viability and proliferation of HDFs on scaffolds. First, punched pieces of each scaffold were placed in a 96-well plate and 1×10^4 ^of the cells cultured in 1 ml of DMEM medium containing 10% FBS were added to each well. After 24, 48, and 72 hr incubation at 37 °C and 5% CO_2_, 50 µl/well of MTT solution was added to all wells, and incubation was carried out for 4 hr. Then, the supernatant was removed and 100 µl of DMSO was added to all wells. After 30 min incubation at room temperature in the dark conditions, the optical density was measured at 570 nm using an ELISA microplate reader (BioTek, USA).

***Antibacterial activity***

To investigate the antibacterial properties of Ben-NPS, standard strains of *Staphylococcus aureus *(ATCC29213) and* Escherichia coli* (ATCC25922) bacteria were kept at 37 °C under constant stirring overnight in the Muller Hinton medium. Next, bacterial suspensions were diluted to 0.5 McFarland (1.5×10^8^ CFU). Two hundred microliters of each bacterial suspension (1.5 × 10^8^ CFU/ml) was spread on Muller Hinton agar plates (6 cm), separately. After that, three circular samples with a diameter of 6 mm were punched from PCL, PCL/CHT, and, PCL/CHT/Ben-NPS fibrous scaffolds and sterilized by 24 hr exposure to UV. Besides, standard disks of vancomycin (30 µg) and gentamycin (10 µg) were utilized as a control for *S. aureus* and *E. coli*, respectively. Then, the plate was kept in an incubator at 37 °C for 24 hr. Eventually, the inhibition zone of each sample was measured and reported.

***Animals***

Forty-five adult male Sprague Dawley rats weighing 180–210 g were purchased from the Center of Comparative and Experimental Medicine at Shiraz University of Medical Sciences, Shiraz, Iran. The animals were maintained under the 12 hr light/dark cycle, at 23±2 °C and 60% humidity. They accessed fresh water and laboratory animals’ standard food pellets freely. They were adapted to the new environment for two weeks before the experiments began. In all experiments, the animals were kept and treated in compliance with the Animal Rights Monitoring Committee of Shiraz University of Medical Sciences (IR.SUMS.MED.REC.1399.182). 

***Experimental design***

We divided the animals into 3 groups randomly (n=15). Deep second-degree burn injury was induced by exposing the dorsal surface of animals’ skin to hot water for ten seconds using a 2 cm firm rubber ring. After 24 hr, the necrotic skin areas were removed and the experimental wounds in each group were dressed with the PCL, PCL/CHT, and PCL/CHT/Ben-NPS nanofibrous scaffolds, respectively.

***Tissue sampling and histopathological evaluations***

In each category five animals were euthanized on days 3, 7, and 14 after burn injury using CO_2_ inhalation. The tissue samples were obtained from the dorsum skin of each rat and fixed in 10% formalin buffer for histopathologic evaluations. Followed by dehydration through a graded series of ethanol embedded in paraffin blocks. The blocks were sectioned at 5–7 μm by a rotary microtome (Leitz 1512, Germany), and prepared slides were stained with hematoxylin and eosin (H&E) stain. After that, the slides were checked with an optical microscope (CX31 Model, Olympus, Japan), and the grade of inflammation, granulation, re-epithelialization, and angiogenesis were quantified for reporting histopathological scores. A score was arbitrarily dedicated (0, absent; 1, scarcely present; 2, present; 3, highly present; 4, intensively present) to each of the above-stated groups to compare them quantitatively (20).

***Statistical analysis***

GraphPad Prism version 9.0.3 (GraphPad Software Inc., San Diego, USA) was used to analyze the data. Data were shown as mean ± standard deviation (SD). ANOVA test followed by *post hoc* Tukey was used to evaluate the significant differences between samples. Data with a *P*-value≤0.05 were statistically significant. 

## Results

***Morphology of scaffolds by sem and diameterj analysis***

**Figure 1:**
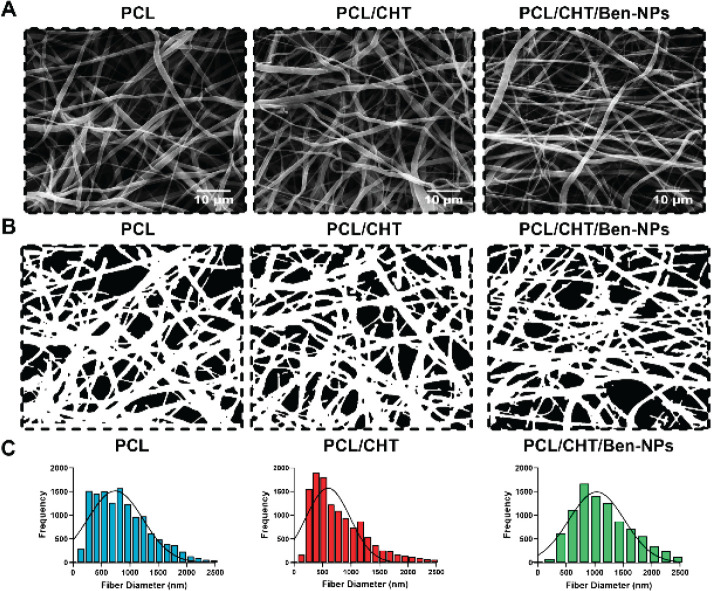
Midsection SEM micrograph, binary image segmentation, and frequency histograms of the PCL, PCL/CHT, and PCL/CHT/Ben-NPS nanofibers (A–C, respectively)

SEM: Scanning electron microscopy; PCL: Polycaprolactone; CHT: Chitosan; Ben-NPS: Bentonite nanoparticles

**Figure 2:**
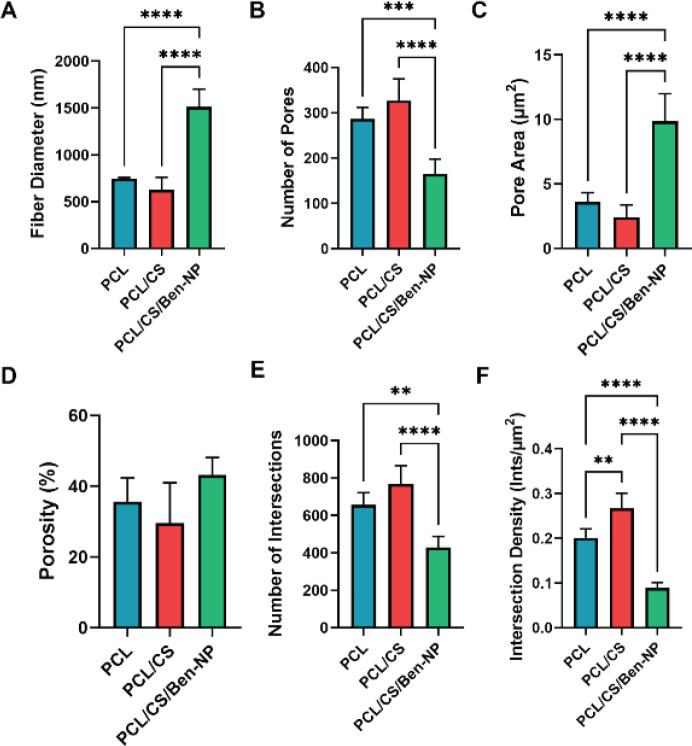
Fiber diameter, porosity, pore area, number of pores, and intersection density (A–F, respectively) in the PCL, PCL/CHT, and PCL/CHT/Ben-NPS nanofibrous scaffolds

The morphology of the scaffolds including PCL, PCL/CHT, and PCL/CHT/Ben-NPS was obtained by SEM (Figure 1A). The micrographs show that fibers in all scaffolds had smooth surfaces without any bead-like formations and were fortuitously distributed throughout the whole scaffold structure. SEM micrographs were segmented with the DiameterJ plugin (Figure 1B) and parameters including fiber radius frequency (Figure 1C), as well as fiber diameter, porosity, intersection, number of pores and intersections, pore area, and density, were obtained (Figure 2A-F, respectively). Results revealed that in the PCL/CHT/Ben-NPS scaffold the fiber diameter was significantly increased (*P*<0.0001 compared to PCL and PCL/CHT scaffolds), and the number of pores was remarkably decreased (*P*<0.001 and *P*<0.0001 compared to PCL and PCL/CHT scaffolds, respectively), and the pore area (*P*<0.0001 compared to PCL and PCL/CHT scaffolds) was significantly increased in comparison to PCL and PCL/CHT scaffolds. Also, the number of intersections and intersection density were significantly reduced in the PCL/CHT/Ben-NPS scaffold compared to the PCL and PCL/CHT fibrous composites. In comparison between electrospun scaffolds, we found no significant difference in terms of the number of pores and porosity.

***FTIR analysis of fibrous scaffolds***

**Figure 3:**
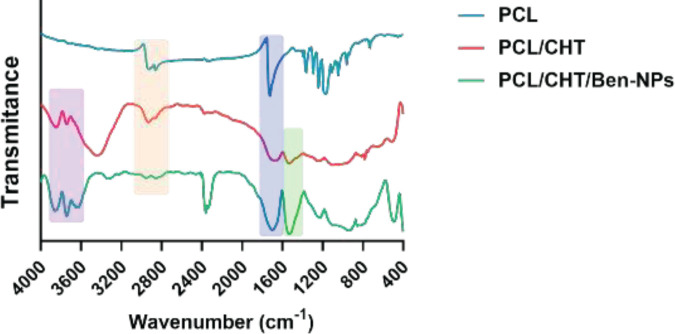
FTIR spectra of the PCL, PCL/CHT, and PCL/CHT/Ben-NPS nanofibers

FTIR: Fourier-transform infrared spectroscopy; PCL: Polycaprolactone; CHT: Chitosan; Ben-NPS: Bentonite nanoparticles

To reveal the chemical content of the electrospun scaffolds FTIR analysis was performed (Figure 3). The FTIR spectrum of the neat PCL scaffold displayed two peaks at 2920 and 2860 cm^-1^ that were consistent with the stretching vibrations of aliphatic C-H bonds. The peak at 1752 cm^-1^ is attributed to the stretching vibration of the carbonyl bond (C=O) of ester groups in PCL. Similar to the raw PCL scaffold, the specific peaks of PCL observed in both PCL/CHT and PCL/CHT/Ben-NPS scaffolds showed similarities along with some extra peaks. The existence of CHT in the PCL/CHT scaffold was confirmed with the broad peak around 3438 cm^-1^ corresponding to the O–H and N–H stretching of CHT (27). N–H bending and C–H stretching vibration were characterized as a fall-off of the amide II at 1528 cm^-1^ (28). In the FTIR spectrum of PCL/CHT/bentonite, the vibration bands at 3615 cm^−1^ and 3406 cm^-1^ stand for O–H stretching and interlayer and intralayer H-bonded O–H stretching, respectively. The bands in the regions 934 and 490 cm^-1^ are related to the stretching and bending vibration of the Si–O group (10, 29-31). In comparison between raw and composite scaffolds, small switches were found due to the molecular interactions between functional groups. 

***Fluid uptake capacity of fibrous scaffolds***

**Figure 4:**
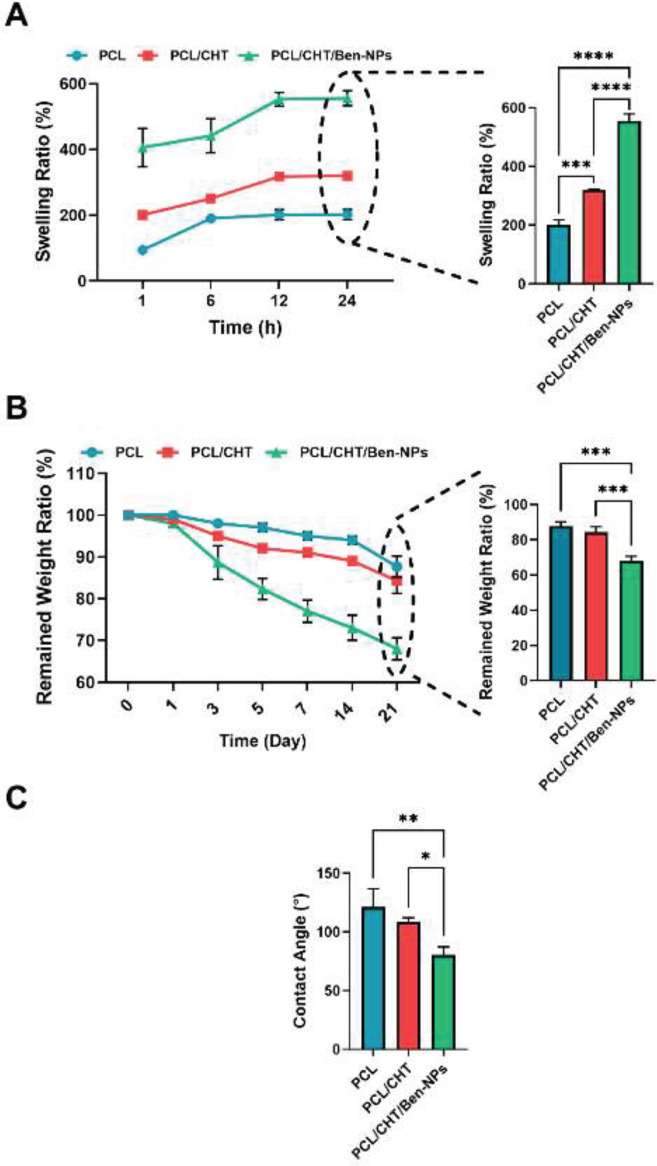
Swelling ratio, remaining weight, and contact angle in the PCL, PCL/CHT, and PCL/CHT/Ben-NPS nanofibrous scaffolds

The swelling ratio in all scaffolds was increased over time until it reached the equilibrium state after 12 hr. The swelling ratios for the PCL, PCL/CHT, and PCL/CHT/Ben-NPS scaffolds after 24 hr were 201.70±16.06 %, 319.90±2.22 %, and 556.20±22.70 %, respectively (Figure 4A). These results indicated that after 24 hr, the swelling ratio was remarkably increased in the PCL/CHT and PCL/CHT/Ben-NPS fibrous composites compared to PCL fibers (*P*<0.01 and *P*<0.001, respectively).

***Degradation ratio in fibrous scaffolds***

The degradation profile of the manufactured nanofibers is presented by the remaining weight ratio (Figure 4B). Results revealed that the remaining weights of the PCL, PCL/CHT, and PCL/CHT/Ben-NPS scaffolds after 1 day were 99.7 ± 0.9, 95 ± 1.3, and 93 ± 1.9%, respectively. After 21 days, the remaining weight of the nanofibers got to 87.66±3.8, 74.33±3.6, and 68.01 ± 1.2%, respectively. The presence of CHT in the structure of the scaffolds showed an improvement in degradation at any time. A significant difference (*P*<0.001) was observed between the degradation of the scaffolds containing CHT and those without CHT after 3 weeks. Additionally, a noticeable effect on the degradation ratio was found when the scaffolds had Ben-NPS. It made a significant difference (*P*<0.0001) at each time.

***Water contact angle in fibrous scaffolds***

We measured the surface wettability of scaffolds by contact angle analysis. The contact angles for PCL, PCL/CHT, and PCL/CHT/Ben-NPS were 121.14 ± 1.12, 108.51 ± 3.1, and 80.32 ± 0.8, respectively (Figure 4C). A significant decrease in contact angle was observed by addition of hydrophilic CHT and CHT/Ben-NPS to the hydrophobic PCL scaffolds (*P*<0.01 and *P*<0.001, respectively). It shows the enhancement of the cell attachments followed by the improvement of the surface hydrophilicity of the nanofibers.

***Mechanical properties of nanofibrous scaffolds***

**Figure 5:**
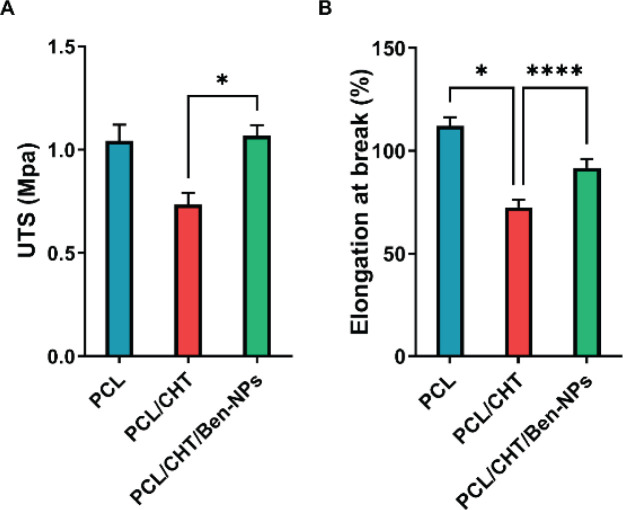
Mechanical properties, including UTS (A) and elongation at break percentage (B) in the PCL, PCL/CHT, and PCL/CHT/Ben-NPS nanofibers

Results indicated that adding CHT to PCL decreased UTS (from 1.04±0.16 MPa to 0.73±0.15 MPa) and a considerable reduction in the elongation at break in comparison to the PCL scaffold (from 112.157±4.14 % to 72.33±3.70 %, *P*<0.05; Figure 5A). Meanwhile, mixing Ben-NPS with PCL and CHT significantly increased UTS (1.07±0.09 MPa, *P*<0.05) and elongation at break (91.65±4.36 %, *P*<0.0001) in the fabricated fibrous nanocomposite in comparison to the PCL/CHT scaffold (Figure 5B).

***Cell viability***

**Figure 6:**
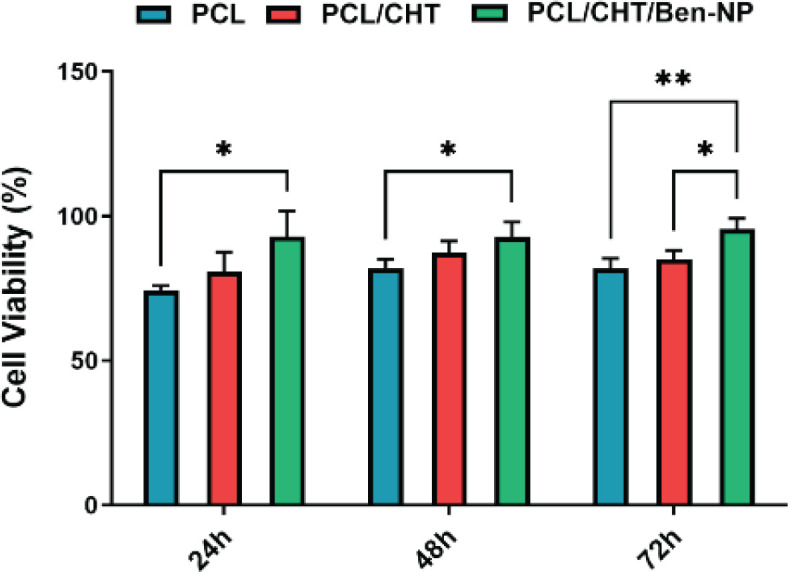
Cell viability percentage of HDFs in the PCL, PCL/CHT, and PCL/CHT/Ben-NPS nanofibers at 24, 48, and 72 hr after cell seeding

MTT assay was utilized to investigate the viability of HDFs on the PCL, PCL/CHT, and PCL/CHT/Ben-NPs fibrous scaffolds (Figure 6). At the time points 24 hr and 48 hr, the viability of HDFs in the PCL/CHT/Ben-NPS group was increased compared to the PCL group (*P*<0.05). At the time point 72 hr, the HDFs had higher viability on the PCL/CHT and PCL/CHT/Ben-NPS scaffolds than on the PCL scaffold (*P*<0.01 and *P*<0.05, respectively).

***Evaluation of cell seeding, migration, and proliferation in the scaffolds by SEM***

**Figure 7:**
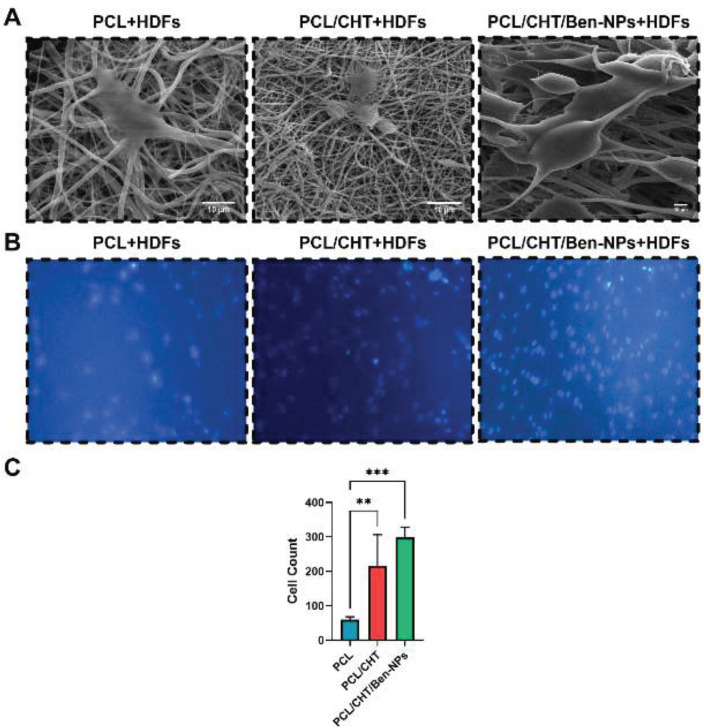
SEM micrographs (A), DAPI staining (B), and number of live cells (C) in the PCL, PCL/CHT, and PCL/CHT/Ben-NPS nanofibers

Results of SEM, DAPI staining, and the number of cells are illustrated in Figure 7A–C, respectively. As is obvious in Figure 7A, HDFs were successfully attached to the fabricated scaffolds. Cell count by ImageJ software indicated that the number of cells on the PCL/CHT (216±90.43) and PCL/CHT/Ben-NPS (298.5±30.38) scaffolds was more significant (*P*<0.01 and *P*<0.001, respectively) compared to PCL (60±8.04). We found no remarkable difference between PCL/CHT and PCL/CHT/Ben-NPs groups (Figure 7C).

***Disk diffusion antibacterial assay***

**Figure 8:**
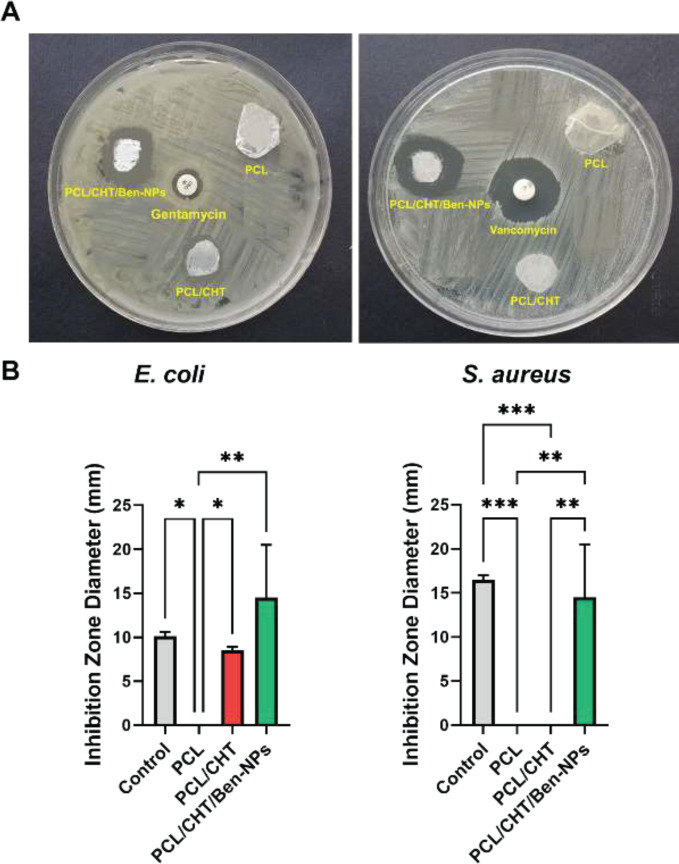
Disk diffusion assay for PCL, PCL/CHT, and PCL/CHT/Ben-NPS against Escherichia coli and Staphylococcus aureus (A). Inhibition zone diameter in different samples (B)

Incorporation of the antibacterial agents into the tissue engineering scaffolds can improve their antimicrobial properties. Based on the disc diffusion results, the PCL/CHT/Ben-NPS scaffold had a positive effect against *E. coli* and *S. aureus* (Figure 8). In fact, for *E. coli*, PCL/CHT/Ben-NPS exhibited a 14.5 ± 0.6 mm inhibition zone while the gentamycin disc indicated a 10.1 ± 0.5 mm inhibition zone diameter. Also, PCL/CHT showed an 8.52±0.47 mm inhibition zone. For *S. aureus*, PCL/CHT/Ben-NPS illustrated a 16.5±0.43 mm inhibition zone while the vancomycin disc exhibited a 21.1 ± 0.35 mm inhibition zone diameter. 

***Macroscopic evaluation and wound closure***

**Figure 9:**
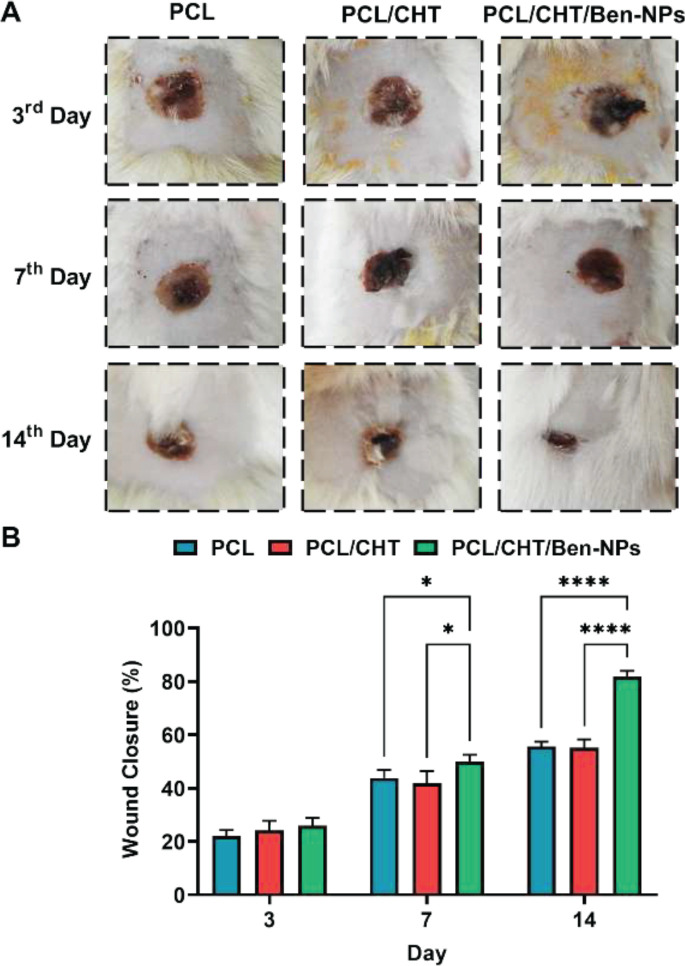
Macroscopic images of skin wounds experimentally created by burn injury in the rat model on days 3, 7, and 14, respectively (A). Wound closure percentage in the animal groups dressed with the PCL, PCL/CHT, and PCL/CHT/Ben-NPS fibrous scaffolds (B)

Macroscopic changes and wound in animal wounds and closure percentage 3, 7, and 14 days after wound dressing are indicated in Figure 9 (A and B, respectively). Our finding indicated that on day 14, the wound closure percentage was more significant in the PCL/CHT/Ben-NPS scaffold than in the PCL/CHT and PCL scaffolds (*P*<0.0001).

***Histopathological evaluations***

**Figure 10:**
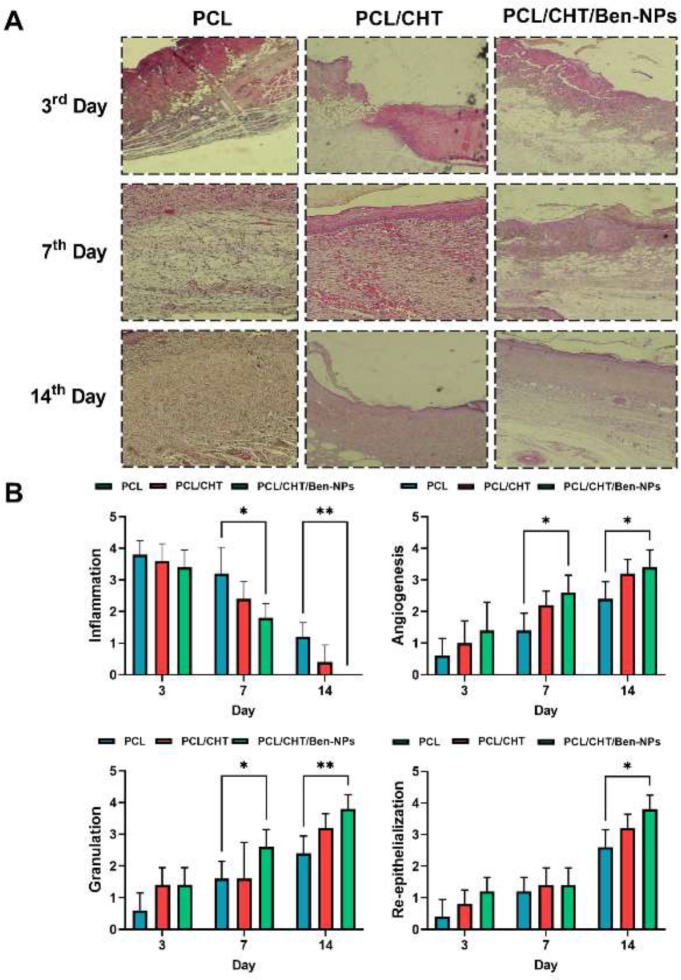
Microscopic aspect of skin wounds experimentally created by burn injury in the rat model (H&E staining) (A) and histopathological scoring of wound samples on days 3, 7, and 14 after dressing with the PCL, PCL/CHT, and PCL/CHT/Ben-NPS fibrous scaffolds, respectively (B–E). Data are indicated as mean ± SD. *P<0.05 and **P<0.01

PCL: Polycaprolactone; CHT: Chitosan; Ben-NPS: Bentonite nanoparticles

Pathological evaluation on day 3 after burn wound induction showed no considerable changes between groups in the repair process. In all groups, the epidermis was destroyed and not regenerated. Also, there was severe vasodilation and a lack of tissue bud formation. In the treatment group tissue buds were forming in the dermis and regeneration of the epidermis had begun as well (Figure 10A). 

On the 7^th^ day after dressing the wound site with the PCL/CHT/Ben-NPS scaffold, the absence of inflammation, bleeding, and infection was seen and there was incomplete tissue repair with mature tissue buds. On day 14, in the PCL/CHT/Ben-NPS group, a complete repair was observed with mature tissue buds, angiogenesis, and normalization of collagen fibers. Inflammatory cells and bleeding were not observed, but in the animals dressed with PCL and PCL/CHT, tissue buds were growing, inflammatory cells and vasodilation were reduced, and tissue normalization was seen (Figure 10A).

On the other hand, histopathological scoring of wound tissue samples indicated that on days 7 and 14, the PCL/CHT/Ben-NPS fibrous scaffold significantly reduced the inflammation score, while it increased angiogenesis and granulation scores in comparison to the PCL scaffold. Also, on day 14, the re-epithelization score remarkably increased in the wound site of animals dressed with the PCL/CHT/Ben-NPS scaffold compared to the PCL (Figure 10B). 

Data are expressed as mean ± SD. *P<0.05, **P<0.01, and **** *P*<0.0001

PCL: Polycaprolactone; CHT: Chitosan; Ben-NPS: Bentonite nanoparticles

Data are expressed as mean ± SD. **P<0.01 and *** *P*<0.001

PCL: Polycaprolactone; CHT: Chitosan; Ben-NPS: Bentonite nanoparticles

Data are expressed as mean ± SD. **P<0.01 and *** *P*<0.001

UTS: Ultimate tensile strength; PCL: Polycaprolactone; CHT: Chitosan; Ben-NPS: Bentonite nanoparticles

Data are expressed as mean ± SD. *P<0.05 and **P<0.01 compared to each other at the same time point.

PCL: Polycaprolactone; CHT: Chitosan; Ben-NPS: Bentonite nanoparticles

Data are indicated as mean ± SD. **P<0.01 and ***P<0.001

PCL: Polycaprolactone; CHT: Chitosan; Ben-NPS: Bentonite nanoparticles

Data are indicated as mean ± SD. *P<0.05, **P<0.01, and ***P<0.001

PCL: Polycaprolactone; CHT: Chitosan; Ben-NPS: Bentonite nanoparticles

Data are indicated as mean ± SD. *P<0.05 and ****P<0.0001

PCL: Polycaprolactone; CHT: Chitosan; Ben-NPS: Bentonite nanoparticles

## Discussion

Nanofibrous-based scaffolds have suitable properties for application as wound dressings due to imitating the fibrous architecture of the ECM (32). In the present study, the blend electrospun nanofibrous scaffold composing PCL and CHT loaded with nanoparticles of Ben, an absorbent swelling clay enriched in montmorillonite, offered cytocompatibility, wettability, and accelerated wound healing as well as antibacterial properties. Hence, this study revealed the benefit of the electrospun PCL/CHT/Ben-NPS scaffold as a promising dressing for deep second-degree burn wounds, considering the findings of *in vitro* and *in vivo* experiments.

SEM micrographs of electrospun nanofibers showed that nanofibers were uniform and well-branched with no visible bead formation. The SEM micrograph of the PCL/CHT/Ben-NPS scaffold did not show any agglomeration of Ben-NPS, which suggests that Ben-NPS can be well-dispersed in the electrospun scaffold. Chakraborty and Pimentel (15) have previously reported easy dispersion of Ben in the CHT matrix and provided an interconnected porous structure scaffold.

Wound dressing scaffolds should have proper mechanical properties in order to support cellular migration and proliferation, as well as subsequent angiogenesis. Moreover, they should protect structures that supply the skin, such as blood and lymphatic vessels, as well as nerve bundles (33). The mechanical properties of the PCL, PCL/CHT, and PCL/CHT/Ben-NPS scaffolds were assessed and results revealed that while UTS did not significantly differ between PCL and two other scaffolds, PCL/CHT/Ben-NPS had remarkably higher UTS than PCL/CHT which probably is due to the interplay between Ben-NPS and PCL/CHT blend and subsequent increase in crystallinity. However, the electrospun scaffolds have not met the tensile strength range of rat skin (1.2–3.2 MPa) (34). Similarly, Barua *et al*. have reported that mixing Ben clay with hyperbranched epoxy reinforced the mechanical properties of the fabricated polymeric nanocomposite (35). The inclusion of nanoclays in a PCL/CHT/Curcumin composite film significantly enhanced the tensile strength (36). Also, the addition of Ben enhanced the tensile strength of a bioplastic based on sago starch (37). 

The scaffolds are required to swell moderately to permit cell proliferation (38), but excessive swelling could lead to the loss of their structural integrity (15). Also, a suitable wound dressing should prevent dehydration and simultaneously have the ability to remove the excessive wound exudate (33). The swelling behavior of the three types of scaffolds revealed that the swelling ratio of the PCL/CHT/Ben-NPS is significantly higher than PCL and PCL/CHT nanofibers. This might be due to its hydrophilic property of Ben. The montmorillonite constituents of Ben have a negative surface charge that allows H_3_O^+^ from the self-ionization of water to easily permeate into the interlayer (39). The swelling capacity of all electrospun scaffolds steadily increased to a certain level following increasing exposure time. After 6 hr in the PCL scaffold and 12 hr for PCL/CHT as well as PCL/CHT/Ben-NPS scaffolds, no considerable increase in swelling was seen which indicates that the fabricated scaffolds could control swelling and, consequently, have a better ability to maintain their structural integrity following exposure to water or body fluid. 

The weight loss ratio over the 21-day *in vitro* incubation period was more significant in the PCL/CHT/Ben-NPS fibrous nanocomposite than the other two nanofibrous scaffolds (PCL and PCL/CHT). This might be due to the biodegradable nature of Ben. According to the current findings, Zamrud *et al*. (37) indicated that loading Ben nanoclays increased the weight loss of a nanocomposite film based on sago starch.

Contact angle measurement can assess the hydrophobic or hydrophilic properties of the fabricated fibrous scaffolds (26). PCL has a hydrophobic attribute that is due to the presence of a five-carbon chain between its ester, which increases the non-polar properties of the material. Hence PCL with hydrophilic natural polymers such as CHT make ideal scaffolds (40). CHT can enhance the wettability and permeability of PCL (41). Water contact angle measurements of electrospun scaffolds showed that although mixing CHT with PCL significantly decreased the amount of the woven scaffold contact angle compared to the electrospun PCL, PCL/CHT scaffold still had more than 90° angle, which indicates hydrophobicity of the scaffold. This might be due to the higher concentration of PCL related to CHT in the sample solution which was 3:1. On the other hand, mixing Ben-NPS with PCL and CHT remarkably lowered the contact angle and thereby became more hydrophilic which can be due to the higher potential of capillary suction and infiltration of water following the addition of Ben-NPS. In line with our results, researchers (42) have indicated PCL/CHT mat included polyamidoamine dendrimer modified montmorillonite nanofibrous intensely decreased water contact angle. Providing an equilibrium aqueous environment for the wound facilitates the wound closure process. Also, more hydrophilic properties of the fabricated scaffold are associated with more cell anchorage (43).

Wound dressing nanofibrous scaffolds must provide a cytocompatible platform for migration, adherence, and proliferation of fibroblasts. Findings from the MTT assay showed that the percentage of cell viability in all electrospun nanofibers seeded with HDFs is acceptable. Therefore, all scaffolds are nontoxic. However, the PCL/CHT/Ben-NPS scaffold showed better results than the others which indicates adding Ben-NPS enhances the cytocompatibility of the scaffold. In another study, different concentrations of Ben in a nanocomposite hybrid hydrogel provided an appropriate environment for cells to grow and proliferate (44).

Cell adhesion to PCL, PCL/CHT, and PCL/CHT/Ben-NPS nanofibers was evaluated by SEM observation. In the PCL/CHT/Ben-NPS scaffold elongated fibroblasts were well-spread throughout the surface of the nanofibrous scaffold while in two other electrospun scaffolds, the cell adhesion was considerably hindered. This evidence could be due to enhancing the surface hydrophilic properties of the PCL/CHT/Ben-NPS nanofiber compared to two other nanofibrous mats. Both CHT and montmorillonite in Ben possess cell adhesive properties (39, 45). Also, an increase in hydrophilicity promotes cell adhesion to the scaffold surface (46).

Disk diffusion antibacterial assay can evaluate the antibacterial activity of the fabricated fibrous scaffolds (47). The result of this experiment revealed that the addition of Ben-NPS to PCL/CHT satisfyingly inhibited *E. coli* and *S. aureus* growth. The bactericidal property of natural clays like Ben is attributed to their ability to induce over-production of hydroxyl radicals via the Fenton reaction which is lethal for bacteria (48). Nabgui *et al*. (14) showed that a bio-composite based on PCL/Ben strongly inhibited the growth of *S. aureus* and *S. epidermis* bacteria. Moreover, a higher solute concentration of Ben in the bio-composite proportionally increased the inhibition zone (14). Researchers (16) have reported the potent antibacterial activity of CHT/Ben composite against *S. aureus* and *P. aeruginosa*. In another study a hyperbranched epoxy-based nanocomposite containing Ben exhibited antimicrobial activity against *S. aureus* and *E. coli* (35) that is similar to our findings. Besides, montmorillonites which are major components of Ben have shown antibacterial activity in several studies (49-51). It has been suggested clay materials can physically adsorb bacterial species and also release anti-bacterial cations (50).

Histopathological evaluations revealed that applying the PCL/CHT/Ben-NPS scaffold facilitated the formation of tissue buds in the dermis and promoted the regeneration of the epidermis. Moreover, after two weeks of dressing with the PCL/CHT/Ben-NPS scaffold, inflammation, bleeding, and infection were eliminated, and incomplete tissue restoration accompanied by mature tissue buds was observed. Eventually, after 21 days, the complete repair with mature tissue buds, angiogenesis, and organization of collagen fibers occurred. In line with our findings, Dastjerdi *et al*. (52) have shown that implantation of a CHT derivative/montmorillonite nanofibrous scaffold on an excisional wound in a rat model leads to complete formation of the epidermal layer and remarkable reduction of the inflammatory cell at the wound site.

## Conclusion

In this research, a novel PCL/CHT-based nanofibrous scaffold reinforced with Ben-NPS was fabricated for application in wound dressing following a deep second-degree burn injury. The proper fabrication of the scaffold was corroborated by SEM imaging. The water contact angle test has shown better surface wettability of the PCL/CHT/Ben-NPS nanofibrous scaffold. Cytotoxicity and cytocompatibility evaluations performed by MTT assay revealed that the PCL/CHT/Ben-NPS nanofibrous scaffold provided the desired environment for the proliferation of HDFs. PCL/CHT/Ben-NPS showed acceptable antibacterial activity. Moreover, the animal study on the burn wound revealed that the application of PCL/CHT/Ben-NPS nanofibrous scaffold improved dermal and epidermal regeneration and decreased inflammation at the wound site. Hence, this nanofibrous scaffold has the potential for dressing skin wounds. 

## Authors’ contributions

SS H, AA M, M G, and B G conceived the study; SS H, AA M, M G, and B G provided methodology; SS H, AA M, and M G performed validation; SS H, A R, and M K helped with formal analysis; SS H, A R, and M K contributed to investigation; SS H, M G, and B G acquired resources; SS H performed data curation; SS H and M K helped with writing and original draft preparation; SS H, A R, and M K helped with writing, review, and editing; SS H, A R, and M K performed visualization; SS H, AA M, and M G provided supervision; SS H, AA M, M G, and B G provided project administration; M G acquired funding. All authors have read and agreed to the published version of the manuscript.

## Institutional Review Bord Statement

The study was approved by the Institutional Ethics Committee of Shiraz University of Medical Sciences (IR.SUMS.MED.REC.1399.182).

## Data Availability Statement

The data that support the findings of this study are available from the corresponding author upon reasonable request.

## Conflicts of Interest

The authors declare no conflicts of interest.
